# Comparative clinical accuracy analysis of the newly developed ZZ IOL and four existing IOL formulas for post-corneal refractive surgery eyes

**DOI:** 10.1186/s12886-021-01991-7

**Published:** 2021-05-25

**Authors:** Jun Zhang, Jie Shao, Li Zheng, Ye Shen, Xia Zhao

**Affiliations:** 1 Ophthalmology, Hangzhou MSK Eye Hospital, Hangzhou, China; 2 Ophthalmology, the First Affiliated Hospital, College of Medicine, Zhejiang University, Hangzhou, China

**Keywords:** Lenses, Intraocular, Refractive surgical procedures, Cataract, Refractive errors

## Abstract

**Background:**

Intraocular lens (IOL) calculation using traditional formulas for post-corneal refractive surgery eyes can yield inaccurate results. This study aimed to compare the clinical accuracy of the newly developed Zhang & Zheng (ZZ) formula with previously reported IOL formulas.

**Study design:**

Retrospective study.

**Methods:**

Post-corneal refractive surgery eyes were assessed for IOL power using the ZZ, Haigis-L, Shammas, Barrett True-K (no history), and ray tracing (C.S.O Sirius) IOL formulas, and their accuracy was compared. No pre-refractive surgery information was used in the calculations.

**Results:**

This study included 38 eyes in 26 patients. ZZ IOL yielded a lower arithmetic IOL prediction error (PE) compared with ray tracing (*P* = 0.04), whereas the other formulas had values like that of ZZ IOL (*P* > 0.05). The arithmetic IOL PE for the ZZ IOL formula was not significantly different from zero (*P* = 0.96). ZZ IOL yielded a lower absolute IOL PE compared with Shammas (*P* < 0.01), Haigis-L (*P* = 0.02), Barrett true K (*P* = 0.03), and ray tracing (*P* < 0.01). The variance of the mean arithmetic IOL PE for ZZ IOL was significantly smaller than those of Shammas (*P* < 0.01), Haigis-L (*P* = 0.03), Barrett True K (*P* = 0.02), and ray tracing (*P* < 0.01). The percentages of eyes within ± 0.5 D of the target refraction with the ZZ IOL, Shammas, Haigis-L, Barrett True-K, and ray-tracing formulas were 86.8 %, 45.5 %, 66.7 %, 73.7 %, and 50.0 %, respectively (*P* < 0.05 for Shammas and ray tracing vs. ZZ IOL).

**Conclusions:**

The ZZ IOL formula might offer superior outcomes for IOL power calculation for post-corneal refractive surgery eyes without prior refractive data.

## Background

Corneal refractive surgery has been used for nearly 30 years, and many treated patients are reaching the age at which senile cataracts might develop [1]. Cataract surgery is a very common surgical procedure [2]. It is expected that patients with a history of corneal refractive surgery will eventually represent an important proportion of the patients with cataracts [3]. While corneal refractive surgery results in excellent uncorrected distance visual activity (UCDVA), it complicates the accurate calculation of the intraocular lens (IOL) power [4, 5].

Many patients who underwent corneal refractive surgery have high requirements for quality of life and high expectations for achieving spectacle independence after cataract surgery. In order to avoid patient dissatisfaction after IOL implantation, achieving the expected diopter (D) is the primary goal [6]. Notwithstanding modern formulas and instruments, calculating IOL power in post-corneal refractive surgery eyes remains a challenging task. In particular, a hyperopic refractive surprise is common in patients with post-myopic corneal refractive surgery [7, 8], which represents the most extensive group of patients with refractive surgery, and seriously affects patient satisfaction.

A number of studies were conducted to select the optimal formula for IOL power calculation. A recent study in patients after corneal refractive surgery revealed that the Barrett True-K formula was superior to the Haigis-L formula [9]. In addition, techniques not requiring prior refraction data (Haigis-L; Shammas; Barrett True-K no-history; Wang-Koch-Maloney; ‘average’, ‘minimum’ and ‘maximum’ IOL power on the ASCRS IOL calculator) showed no significant differences in IOL PE for post-LASIK eyes [10]. A robust study assessing the accuracy of 12 IOL power formulas and revealed that the Olsen and Barrett formulas have excellent accuracy for overall eyes [11]. However, traditional empirical formulas are mainly based on statistical regression methods, making it difficult to calculate IOL power accurately. There are two major causes of error in IOL calculation for post-corneal refractive surgery eyes: (1) incorrect corneal refractive power estimation [12, 13]; and (2) incorrect effective lens position (ELP) estimation [14, 15]. Both problems are related to the empirical formula of irregular cornea deviation. Some formulas also attempt to circumvent the above issues using historical data (e.g., the Double K [8], Diehl-Miller nomogram [16], and Feiz-Mannis formula [17]), but the proportion of patients with available historical data is limited, and the outcome is also affected by multiple factors such as historical data accuracy, corneal curvature changes caused by the relaxation of orbicularis oculi muscle, and eye axis growth. Therefore, these methods cannot solve the two issues above [18]. In order to avoid these two issues, IOL calculation based on Snell’s law and ray tracing might be a good option because the ray tracing algorithm is based on the actual refractive state of the cornea [19] and could relatively reduce the error source of corneal refractive power.

We recently developed the Zhang & Zheng IOL power formula (ZZ IOL), for which a patent application has been filed (application number: 2,019,100,304,230 on the Chinese invention patent website, www.cpquery.sipo.gov.cn). ZZ IOL can be accessed at www.zzcal.com. We hypothesized that ZZ IOL is more accurate in calculating IOL power compared with existing traditional formulas. The purpose of this study was to compare the clinical accuracy of ZZ IOL and formulas on the ASCRS post-refractive IOL calculator and the ray tracing method.

## Methods

### Study design and patients

This retrospective study included patients with a history of corneal refractive surgery and eventually underwent cataract surgery between February 2018 and April 2021 at the First Affiliated Hospital to the College of Medicine of Zhejiang University.

The inclusion criteria were 1) ≥ 18 years of age, 2) a history of phacoemulsification cataract surgery and primary in-the-bag implantation of posterior chamber IOL, 3) corneal refractive surgery before the cataract surgery, and 4) manifest refractive spherical equivalent (MRSE) applied at 1 month after cataract surgery. The exclusion criteria were (1) complications during or after the cataract surgery, (2) corneal sutures, (3) corneal astigmatism > 1.50 D, (4) lens nucleus opacity that might interfere with ocular biometry measurements, or (5) neurological condition that might interfere with the performances of the required tests.

 This study was approved by the Institutional Review Board of the First Affiliated Hospital to the College of Medicine of Zhejiang University [MSKLL20190505]. All procedures adhered to the Declaration of Helsinki. The requirement for informed consent was waived because of the retrospective nature of the study.

### Data collection

All patients underwent complete ophthalmic examinations, including UCDVA (Snellen lines), spherical equivalent (SE), slit-lamp microscopy, intraocular pressure (IOP) measurement, anterior segment tomography (Sirius; CSO, Florence, Italy), biometry (IOLMaster 700; Carl Zeiss, Jena, Germany), and optical coherence tomography (OCT) (Cirrus HD-OCT 5000; Carl Zeiss, Jena, Germany). Anterior segment tomography was performed by an independent physician. Each eye had at least three measurements with good reproducibility. If the breakup time of tear film (BUT) was too short to allow a good measurement, artificial tear drops were used, and BUT was measured 10 min later.

According to whether the cornea had a radial scar and whether the central corneal thickness was significantly thinner than the surrounding area, the previous corneal refractive operation method could be determined, including laser-assisted in situ keratomileusis (LASIK), radial keratotomy (RK), and RK + LASIK. The reference information was obtained through slit lamp, corneal tomography, and OCT examination.

### Surgery

All cataract surgeries were performed by the same surgeon (SY). After topical anesthesia (Alcaine; Alcon, Fort Worth, TX, USA), capsulorhexis, lens fragmentation, and double incisions (main incision and lateral incision) were performed with a femtosecond laser (LenSx; Alcon, Fort Worth, TX, USA). The main clear corneal incision was in the axis of the steep corneal curvature. After the femtosecond laser procedure, the surgeon opened two incisions with a bladeless hook and injected ophthalmic viscoelastic devices (OVDs) (DisCoVisc; Alcon, Fort Worth, TX, USA) to maintain the anterior chamber. Then, free-floating anterior capsule removal was performed, followed by cortical-cleaving hydrodissection and the stop-and-chop technique for phacoemulsification. The irrigation-aspiration (I/A) probe was used for cortical removal and polishing, and the IOL was implanted into the capsular pouch directly via the 2.2-mm main incision (Centurion; Alcon, Fort Worth, TX, USA). IOL power was selected according to the ZZ IOL formula. The type of IOL (AT LISA tri 839MP, AT LISA 809MP, or CT ASPHINA 509MP, Carl Zeiss, Table 1) was selected based on anterior segment tomography. After OVDs removal with the I/A probe, stromal hydration was used to close the incisions. Postoperative eyedrops included topical antibiotics and corticosteroids (levofloxacin, Santen, Japan, and Tobradex, Alcon, Fort Worth, TX, USA).

**Table 1:** The IOLs used in the included patients

	AT LISA tri 839MP	AT LISA 809MP	CT ASPHINA 509MP
Clinical	Tri-focal IOL	Bi-focal IOL	Mono-focal IOL
Optical	+ 3.33 D for near focus and + 1.66 D for intermediate focus	+ 3.75 D for near focus	No additional diopter
Advantages	Can assist in near and intermediate visual acuity	Can assist in near visual acuity	High contrast sensitivity; suitable for patients with corneal irregularity
Disadvantages	Not suitable for patients with corneal irregularity	Visual acuity might be slightly affected by corneal irregularity	No assistance in near or intermediate visual acuity
Manufacturer	Carl Zeiss (Jena, Germany)	Carl Zeiss (Jena, Germany)	Carl Zeiss (Jena, Germany)

*IOL* intraocular lens, *D* diopters

### The ZZ IOL formula

The ZZ IOL formula considers three major refractions (including the anterior corneal surface, posterior corneal surface, and IOL) in the process of focusing the incident ray on the retina. The IOL was modeled as a thin lens. The anterior corneal tangential curvature (K_f_), central corneal thickness (CCT), posterior corneal tangential curvature (K_b_), and anterior chamber depth (ACD) were obtained by anterior segment tomography. Notably, K_f_ and K_b_ were the average curvatures of the anterior and posterior corneal surfaces of the effective optical zone which was determined by the physician. The axial length (AL) and lens thickness (LT) were obtained by biometry. Lens constants referred to the User Group for Laser Interference Biometry website (ULIB, www.ocusoft.de/ulib/).

The distance S_1_ from the incident ray source to the anterior corneal surface was calculated using the target D (D_t_) after cataract surgery (Eq. 1).

1 $${S}_{1}\approx -\frac{1}{\frac{{D}_{t}}{1-0.012{D}_{t}}-0.0001}$$

where “-0.0001” must be added to avoid a denominator of 0 and an infinite S_1_ value.

The selection of K_f_, CCT, and K_b_ was based on the lower curvature region around the vertex in the refractive equivalent power map, which was obtained from Sirius. The refractive equivalent power map was expressed in D and calculated by ray tracing through the anterior and posterior corneal surfaces for each point. The reference indices for the two interfaces were the air index (N_1_ = 1), stroma index (N_3_ = 1.376), and an index for the aqueous humor (N_4_ = 1.336).

The distance S_2_ from the image focus of the incident ray source to the anterior corneal surface after the first refraction was calculated (Eq. 2).

2 $${S}_{2}=\frac{{N}_{2}}{\frac{\left({N}_{2}-{N}_{1}\right){K}_{f}}{1.3775-{N}_{1}}-\frac{{N}_{1}}{{S}_{1}}}$$

where N_2_ = 1.376 for the corneal index.

The distance S_3_ from the image focus of the incident ray source to the posterior corneal surface after the second refraction was calculated (Eq. 3).

3 $${S}_{3}=\frac{{N}_{3}}{-{K}_{b}-\frac{{N}_{2}}{{Z}_{1}-{S}_{2}}}$$

where Z_1_ is related to CCT. The purpose of introducing Z_1_ is to determine the approximate image focus of a thick lens using a formula for a thin lens.

The distance (S_4_) from the image focus of the incident ray source to the IOL after the third refraction was calculated (Eq. 4).

4 $${S}_{4}=\frac{AL}{1000}-\frac{CT}{1,000,000}-\frac{AC}{1000}-{Z}_{2}$$

where Z_2_ is related to LT and IOL lens constant. The purpose of introducing Z_2_ is to determine the distance between the anterior capsule and the IOL.

The ZZ IOL-predicted power was calculated according to S_3_ and S_4_ (Eq. 5).

5 $$IOL power=\frac{\left(\frac{{N}_{3}}{{Z}_{3}-{S}_{3}}+\frac{{N}_{3}}{{S}_{4}}\right)}{{Z}_{4}}$$

where Z_3_ is related to AC + Z_2_. Z_3_ was introduced for the same purpose as Z_1_, with a similar formula. Z_4_ is the IOL index used in the ZZ IOL formula.

### Standard IOL formulas

Based on biometric data (including curvatures of flat axis and steep axis, AL, ACD, LT, and white to white [WTW]) obtained before cataract surgery, the ASCRS post-refractive IOL calculator was used to assess IOL power using Haigis-L [20], Shammas [21], and Barrett True-K (no history) [22]. No pre-corneal refractive surgery information was used in any of the calculations. Based on anterior segment tomography data, the Sirius internal IOL calculator was used to obtain the IOL power of ray tracing [23].

### IOL prediction error

The manifest refractive SE was obtained 1 month after cataract surgery. The predicted IOL power for each formula was calculated by targeting the actual SE following cataract surgery, and the IOL prediction error (PE) was then calculated (Eq. 8) [24].

8 $$\text{I}\text{O}\text{L} \text{P}\text{E}=\text{I}\text{m}\text{p}\text{l}\text{a}\text{n}\text{t}\text{e}\text{d} \text{I}\text{O}\text{L} \text{P}\text{o}\text{w}\text{e}\text{r}-\text{P}\text{r}\text{e}\text{d}\text{i}\text{c}\text{t}\text{e}\text{d} \text{I}\text{O}\text{L} \text{P}\text{o}\text{w}\text{e}\text{r}$$

A positive value indicated that the method predicted an IOL of lower power than the implanted IOL, leaving the patient hyperopic. Mean arithmetic and absolute IOL PEs and variances of the mean were calculated.

### Refractive prediction error

Using the assumption that an IOL PE of 1.00 D produces 0.70 D of refractive error at the spectacle plane, the absolute refractive PEs and the percentages of eyes with a refractive PE within ± 0.50 D and ± 1.00 D were calculated for each formula.

### Statistical analysis

Continuous variables were tested for distribution normality by the Kolmogorov-Smirnov test, and homoscedasticity was determined by Levene’s test. Those with a normal distribution are presented as means ± standard deviations (SD) (ranges were also provided). Arithmetic and absolute IOL PEs, variances of mean arithmetic IOL PE, and absolute refractive PEs are expressed as means ± SD and medians (quartiles). Categorical variables are expressed as numbers and percentages. Of all parameters, only the arithmetic PE of ZZ IOL had a normal distribution. Therefore, the one-sample t-test was used to compare the arithmetic PE of ZZ IOL data with a target of zero, and Friedman’s test was used to compare ZZ IOL with the other four formulas. The Wilcoxon signed-rank test was used for the comparison of two related samples. After chi-square segmentation, McNemar’s paired chi-square test was performed to assess the percentages of refractive PE within ± 0.50 D and ± 1.00 D, as well as for multiple group comparisons. The Bonferroni method was used for pairwise comparisons. All statistical analyses were performed using SPSS 19.0 (IBM, Armonk, NY, USA). Two-sided *P* < 0.05 was considered statistically significant.

## Results

### Characteristics of the patients

Patient characteristics are shown in Table 2. Thirty-eight eyes in 26 patients were included. The patients were 56.7 ± 8.0 years old, ranging from 41 to 72 years, and included eight males. Of the 38 eyes, 33 (86.8 %), three (7.9 %), and two (5.3 %) underwent LASIK, RK, and RK + LASIK, respectively. Concerning the IOL types, 21 (55.3 %), eight (21.1 %), and nine (23.7 %) eyes received AT LISA trifocal 839MP, AT LISA bifocal 809MP, and CT ASPHINA 509MP IOLs, respectively.

**Table 2:** Patient characteristics

Variable	N	Mean ± SD/%	Range	
Age (years)	26	56.7 ± 8.0	41 − 72	
Sex (male)	8	30.8 %	-	
Eye used				
Right	19	50.0 %	-	
Left	19	50.0 %	-	
K_f_	38	35.27 ± 3.21	28.37 − 40.85	
K_b_	38	-6.08 ± 1.03	-8.10−-3.49	
Central corneal thickness (mm)	38	472.37 ± 36.00	417 − 590	
Axial length (mm)	38	27.61 ± 2.28	24.85 − 32.26	
Anterior chamber depth (mm)	38	3.12 ± 0.36	2.17 − 3.90	
Lens thickness (mm)	38	4.49 ± 0.42	3.98 − 5.49	
White to white (mm)	38	11.80 ± 0.41	11.00 − 12.60	
Corneal refractive surgery				
LASIK	33	86.8 %	-	
RK	3	7.9 %	-	
RK + LASIK	2	5.3 %	-	
Type of IOL				
AT LISA tri 839MP	21	55.3 %	-	
AT LISA 809MP	8	21.1 %	-	
CT ASPHINA 509MP	9	23.7 %	-	
IOL power (D)	38	19.74 ± 3.99	12.50 − 28.00	

*K*_f_ the anterior corneal tangential curvature, *K*_b_ posterior corneal tangential curvature, *LASIK* laser-assisted in situ keratomileusis, *RK* radial keratotomy, *IOL* intraocular lens, *D* diopters

### Mean arithmetic and absolute IOL PEs

**Fig. 1:**
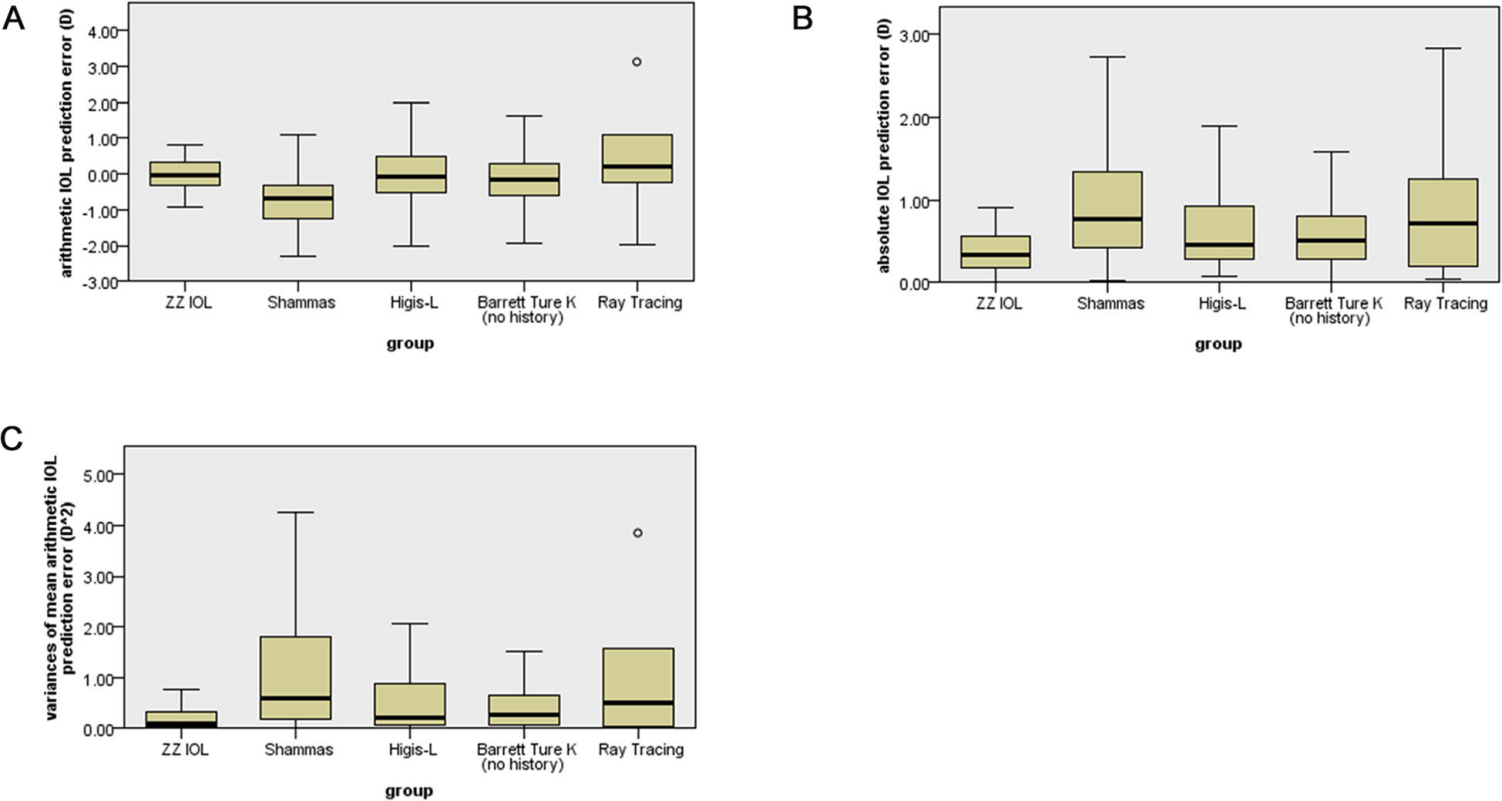
Box diagram of IOL prediction errors. **a** Arithmetic IOL prediction error. **b** Absolute IOL prediction error. **c** Variance of mean arithmetic IOL prediction error

The mean arithmetic and absolute IOL PEs and variances of mean arithmetic IOL PEs are shown in Table 3; Fig. 1. ZZ IOL yielded a lower arithmetic IOL PE compared with ray tracing (*P* = 0.04), whereas the other formulas had values like that of ZZ IOL (*P* > 0.05). In addition, the arithmetic IOL PE for the ZZ IOL formula was not significantly different from zero (*P* = 0.96). ZZ IOL yielded a lower absolute IOL PE compared with Shammas (*P* < 0.01), Haigis-L (*P* = 0.02), Barrett true K (*P* = 0.03), and ray tracing (*P* < 0.01). The variance of the mean arithmetic IOL PE for ZZ IOL was significantly smaller than those of Shammas (*P* < 0.01), Haigis-L (*P* = 0.03), Barrett True K (*P* = 0.02), and ray tracing (*P* < 0.01).

**Table 3:** Arithmetic and absolute IOL PEs (implanted IOL power - predicted IOL power), and variances of mean arithmetic IOL PE

Formula	N	Arithmetic IOL PE (D)		Absolute IOL PE (D)		Variances (D^2^)
		Mean ± SD	Median (Quartile )	Mean ± SD	Median (Quartile)	Median (Quartile)
ZZ IOL	38	-0.00 ± 0.47	-0.02 (-0.33 to 0.35)	0.38 ± 0.26	0.33 (0.18 to 0.57)	0.11 (0.03 to 0.33)
Shammas	33^a^	-0.40 ± 1.84	-0.69 (-1.23 to -0.31)	1.18 ± 1.46	0.77 (0.42 to 1.34)^b^	0.60 (0.18 to 1.81)^b^
Haigis-L	33^a^	0.21 ± 2.07	-0.08 (-0.52 to 0.48)	1.09 ± 1.76	0.46 (0.29 to 0.93)^b^	0.22 (0.08 to 0.87)^b^
Barrett True K (no history)	38	0.19 ± 1.98	-0.16 (-0.59 to 0.30)	1.04 ± 1.68	0.52 (0.28 to 0.80)^b^	0.27 (0.08 to 0.65)^b^
Ray tracing	38	0.65 ± 1.97	0.20 (-0.24 to 1.11)^b^	1.10 ± 1.76	0.71 (0.20 to 1.25)^b^	0.51(0.04 to 1.57)^b^

*IOL* intraocular lens, *D* diopters

^a^No IOL calculation results obtained with Shammas and Haigis-L formula in one eye since post-radial keratotomy was not available

^b^Significantly different versus ZZ IOL (*P* < 0.05, with Bonferroni correction)

### Frequencies of refractive PEs within ± 0.50 and ± 1.00 D

The mean absolute refractive PEs and the percentages of eyes with refractive PEs within ± 0.50 and ± 1.00 D are shown in Table 4. The absolute refractive PE for ZZ IOL was significantly lower than those of Shammas (*P* < 0.01), Haigis-L (*P* = 0.02), Barrett true K (*P* = 0.04), and ray tracing (*P* < 0.01). In addition, ZZ IOL had a significantly greater percentage of eyes within ± 0.50 D of refractive PE compared with Shammas (*P* < 0.01) and ray tracing (*P* < 0.01). Meanwhile, all eyes had a refractive PE within ± 1.00 D for the ZZ IOL formula.

**Table 4:** Absolute refractive PEs and the numbers and percentages of eyes with refractive PE within ± 0.50 and ± 1.00 D

Formula	N	Absolute refractive PE		± 0.50 D	± 1.00 D
		Mean ± SD	Median (Quartile)	N (%)	N (%)
ZZ IOL	38	0.27 ± 0.18	0.24 (0.13 to 0.40)	33 (86.8 %)	38 (100.0 %)
Shammas	33^a^	0.83 ± 1.02	0.54 (0.29 to 0.94)^b^	15 (45.5 %)^b^	26 (78.8 %)
Haigis-L	33^a^	0.76 ± 1.23	0.33 (0.20 to 0.65)^b^	22 (66.7 %)	27 (81.8 %)
Barrett True K (no history)	38	0.73 ± 1.18	0.36 (0.20 to 0.57)^b^	28 (73.7 %)	32 (84.2 %)
Ray tracing	38	0.77 ± 1.23	0.50 (0.14 to 0.88)^b^	19 (50.0 %)^b^	32 (84.2 %)

*IOL* intraocular lens, *D* diopters

^a^No IOL calculation results obtained with Shammas and Haigis-L formula in five eyes since post-radial keratotomy was not available

^b^Significantly different versus ZZ IOL (*P* < 0.05, with Bonferroni correction)

## Discussion

Corneal refractive surgery complicates IOL power calculation for cataract surgery [6–8]. Formulas are available but associated with an inaccuracy of ± 0.5 D [21, 25, 26], leading to patient dissatisfaction. This study aimed to compare the clinical accuracy of the ZZ IOL formula with those of previously developed formulas and methods. The results suggested that the ZZ IOL formula might offer superior outcomes in IOL power calculation for eyes with previous corneal refractive surgery without prior refractive data.

Cataract surgery outcomes include more than simple visual restoration. The patients wish to improve their quality of life by achieving spectacle independence, especially individuals who solicited post-corneal refractive surgery technologies for quality of life improvement. Notwithstanding modern technology and materials, spectacle independence for all distances after cataract surgery remains a huge challenge [27]. Multifocal IOL is a major modality used to achieve this goal [6]. Selecting a proper IOL is an important factor in improving patient satisfaction, not only the model but also its power.

Incorrect corneal refractive power estimation and incorrect ELP estimation are two major causes of refractive error. Various factors can lead to corneal refractive power errors. First, traditional IOL power calculators, for normal or irregular cornea, use a fixed ratio to determine the posterior corneal refractive power, and different calculators have different fixed values [28, 29]. Corneal refractive power bias will be positively correlated with the ratio bias, which is significant for an irregular cornea. This should be the main reason why formulas for post-corneal refractive surgery need to be used according to the classification of corneal refractive surgery. In order to avoid this error, the actual posterior corneal refractive power must be used instead of calculation data. Modern corneal tomography provides accurate and reproducible estimates of the anterior and posterior corneal refractive power, but determining the refractive power for calculation remains a challenge, compounded by no available formula developed to date. Secondly, in traditional IOL power calculators, K1 and K2 represent the mean values of two keratometry readings, symmetrical to the corneal apex, on the flattest and steepest axes, respectively. Certainly, this method is feasible for a normal cornea with small high-order aberrations (HOAs), but an irregular cornea with high HOAs would have larger errors. In addition, corneal asphericity plays a role in the error, and all HOAs have certain effects [30]. In order to avoid the complex aberration problem and reduce this error, anterior and posterior corneal refractive power selection assisted by a corneal refractive equivalent power map should be performed. Thirdly, like the above situation, the more deviation is present, the more error induced. Although this error is hardly greater than 0.10 D, introducing CCT should also be beneficial. In terms of ELP, errors may be introduced because of the use of corneal power to predict ELP (such as Hoffer Q, Holladay 1, and SRK/T), the use of ACD and AL to predict ELP (such as Haigis et al. [31]), the use of pre-refractive surgery K to predict ELP (such as double-K [32]), and the use of ACD and anterior chamber angle to predict ELP (such as ray tracing). Indeed, all these methods are similar and based on some standard coefficients through regression calculation. Of course, the actual ELP cannot be obtained before the operation, but modern partial coherence interferometry can determine the ACD and thickness of the crystalline lens [14]. The IOL would be implanted into the crystalline lens capsule. Then, the effective IOL foot position could be calculated by LT and a specific coefficient. The distance from the optical plane of effective IOL to the foot plane of effective IOL could be calculated by lens constant. Therefore, we consider that the ELP obtained by the sum of these two could introduce a smaller error due to fewer variables and narrower ranges of variables. Based on the above considerations and Snell’s law, the ZZ IOL formula was designed. The main optical theory of ZZ IOL is as follows. First, the effective optical zone was determined by a corneal refractive equivalent power map, which could be obtained by a corneal tomography device. The selection of the effective optical zone mainly depends on two points: the zone within 4.0 mm around the corneal apex and the relatively concentrated focus zone after parallel light passing through the cornea. Second, the mean values of the corneal anterior surface curvature, posterior surface curvature, and thickness in the effective optical zone are used to participate in the ray tracing calculation, i.e., the first and second refraction of the incoming light. Third, ELP was estimated by anterior chamber depth plus the percentage of crystalline lens thickness. Finally, according to the outgoing light after the second refraction, ELP, and the outgoing light of the third refraction focus on the retina, i.e., the end of the ocular axis, the refractive power required for the third refraction can be calculated, i.e., the IOL power.

The ZZ IOL formula might have some advantages over the existing methods. Theoretically, ZZ IOL could be used in a wide range of patients and is not limited by the AL or the type of pre-refractive surgery. In this study, only post-corneal refractive surgery eyes were included to compare its clinical accuracy with formulas on the ASCRS post-refractive IOL calculator and ray tracing. According to the above results, ZZ IOL might show good predictability and accuracy for post-corneal refractive surgery eyes, even in post-RK and post-RK + LASIK eyes. We did not compare the previous formulas among themselves, solely focusing in this study on ZZ IOL comparison with individual formulas, respectively. Concerning the absolute IOL PE, the variances of arithmetic IOL PE and refractive PE, ZZ IOL showed a significant advantage over the Shammas, Haigis-L, Barrett true K, and ray tracing formulas. Future studies with larger sample sizes are required to confirm these findings. In addition, ZZ IOL does not require clinical history information, which can be easily lost during the decades separating corneal refractive surgery from cataract surgery. For corneal conditions/procedures without specific IOL formulas, including keratoconus, post-corneal trauma, post-keratoplasty, and post-stromal ring implantation, ZZ IOL may also be a good option. Normal cornea, irregular cornea, and AL will be examined in future studies. Evaluating the effects of ACD and lens thickness (LT) on nine IOL power calculation formulas accuracy in individuals with normal axial lengths, it was found that new generation formulas, particularly Kane, PEARL-DGS (Prediction Enhanced by Artificial Intelligence and Output Linearization) and Emmetropia Verifying Optical (EVO) formula 26 (version 2.0), have higher reliability and stability even in eyes with extreme ACD-LT combinations [33]. Furthermore, the improvement of post-IOL refractive accuracy can provide doctors with more confidence for operating on post-corneal refractive surgery patients, reduce the proportion of refractive enhancement surgery, and might help more people enjoy the advantages of multifocal IOL in daily life.

However, ZZ IOL also has some disadvantages. Indeed, it is greatly affected by the survey quality of cornea tomography. For major corneal curvature data, Sirius does not seem reproducible as the IOL master [34]. Poor repeatability of corneal tomography would probably make ZZ IOL-derived data fluctuate greatly. In addition, the relatively poor tear film quality of the elderly might reduce repeatability. In order to reduce this error, some methods can be considered. First, the mean or median value of multiple measurements could be used. Second, anterior-segment OCT could be used instead of traditional corneal tomography; the higher resolution, higher definition, and relatively higher quality of anterior segment scans may produce better results [35]. Third, Placido-disk, Scheimpflug, and anterior-segment OCT protocols could all be used to measure the corneal curvature, but each has its advantages [36]. For a specific cornea, choosing the appropriate measurement technology could provide real results. For example, the Sirius device, which combines a rotating Scheimpflug camera with Placido-disk topography, could have issues with some corneas with poor transparency, which causes serious recognition errors in Scheimpflug raw images. Mechanical instability of the cornea following incisional surgery is another influencing factor. Phacoemulsification may partially reopen keratorefractive incisions [37] and cause varying central flattening and peripheral bulging degrees. Fortunately, no significant abnormality was found in both cases included in the present study, but additional patients are needed to draw firm conclusions.

This study had several limitations. First, the sample size was relatively small, with all patients treated in a single center and selected based on specific criteria. In these conditions, selection and information biases could not be avoided. Analyses specific to RK could not be performed. Second, because of the limitation of the number of samples, we included three kinds of IOLs in the study, including multifocal and bifocal IOLs. Although we only evaluated the diopter of the distant focus, the difference in the optical properties of the IOLs may also lead to deviation. Third, the study was retrospective, with inherent shortcomings. Fourth, the same surgeon performed all procedures, and all lenses were produced by the same manufacturer, further indicating possible selection bias. Fifth, only Chinese eyes were included, and high myopia and long AL are highly prevalent in the Chinese population, affecting IOL power calculation [38]. Therefore, this method undoubtedly requires further refinement and additional testing.

## Conclusions

In conclusion, the ZZ IOL formula has reduced mean absolute IOL, and refractive PE compared with the Shammas, Haigis-L, Barrett true K, and ray tracing formulas and shows good accuracy and predictability in post-corneal refractive surgery eyes. Future studies comparing ZZ IOL and other formulas in various types of eyes are warranted.

## Data Availability

The datasets used and/or analysed during the current study are available from the corresponding author on reasonable request.

## Abbreviations

ZZIOL: Zhang & Zheng IOL
PE: Prediction error
D: Diopter
UCDVA: Uncorrected distance visual activity
IOL: Intraocular lens
ASCRS: AmericanSociety of Cataract and Refractive Surgery
ELP: Effective lens position
MRSE: Manifest refractive spherical equivalent
SE: Spherical equivalent
IOP: Intraocular pressure
OCT: Optical coherence tomography
BUT: Breakup time oftear film
SY: All cataract surgeries were performed by one surgeon
CCT: Central corneal thickness
AC: Anterior chamber depth
AL: Axial length
LT: Lens thickness
ULIB: User Group for Laser Interference Biometry
SD: Standarddeviation
LASIK: Laser-assisted in situ keratomileusis
RK: Radial keratotomy

## References

[1] Klein BE, Klein R, Lee KE, Gangnon RE (2008). Incidence of age-related cataract over a 15-year interval the Beaver Dam Eye Study. Ophthalmology.

[2] Liu YC, Wilkins M, Kim T, Malyugin B, Mehta JS (2017). Cataracts. Lancet.

[3] Patel RH, Karp CL, Yoo SH, Amescua G, Galor A (2016). Cataract Surgery After Refractive Surgery. Int Ophthalmol Clin.

[4] Gimbel HV, Sun R (2001). Accuracy and predictability of intraocular lens power calculation after laser in situ keratomileusis. J Cataract Refract Surg.

[5] Koch DD, Liu JF, Hyde LL, Rock RL, Emery JM (1989). Refractive complications of cataract surgery after radial keratotomy. Am J Ophthalmol.

[6] Alio JL, Plaza-Puche AB, Fernandez-Buenaga R, Pikkel J, Maldonado M (2017). Multifocal intraocular lenses: an overview. Surv Ophthalmol.

[7] Seitz B, Langenbucher A, Nguyen NX, Kus MM, Kuchle M (1999). Underestimation of intraocular lens power for cataract surgery after myopic photorefractive keratectomy. Ophthalmology.

[8] Odenthal MT, Eggink CA, Melles G, Pameyer JH, Geerards AJ, Beekhuis WH (2002). Clinical and theoretical results of intraocular lens power calculation for cataract surgery after photorefractive keratectomy for myopia. Arch Ophthalmol.

[9] Wang XZ, Cui R, Song XD, Yun B, Qian J, Ding N (2020). Comparison of the accuracy of intraocular lens power calculation formulas for eyes after corneal refractive surgery. Ann Transl Med.

[10] Patel P, Ashena Z, Vasavada V, Vasavada SA, Vasavada V, Sudhalkar A, et al. Comparison of intraocular lens calculation methods after myopic laser-assisted in situ keratomileusis and radial keratotomy without prior refractive data. Br J Ophthalmol. 2020:bjophthalmol-2020-317681:1–7. 10.1136/bjophthalmol-2020-317681 33093154

[11] Rocha-de-Lossada C, Colmenero-Reina E, Flikier D, Castro-Alonso FJ, Rodriguez-Raton A, Garcia-Madrona JL, et al. Intraocular lens power calculation formula accuracy: Comparison of 12 formulas for a trifocal hydrophilic intraocular lens. Eur J Ophthalmol. 2020:1120672120980690:1–8. 10.1177/1120672120980690 33339479

[12] Hoffer KJ (2009). Intraocular lens power calculation after previous laser refractive surgery. J Cataract Refract Surg.

[13] Langenbucher A, Haigis W, Seitz B (2004). Difficult lens power calculations. Curr Opin Ophthalmol.

[14] Martinez-Enriquez E, Perez-Merino P, Duran-Poveda S, Jimenez-Alfaro I, Marcos S (2018). Estimation of intraocular lens position from full crystalline lens geometry: towards a new generation of intraocular lens power calculation formulas. Sci Rep.

[15] Pinero DP, Camps VJ, Ramon ML, Mateo V, Perez-Cambrodi RJ (2015). Error induced by the estimation of the corneal power and the effective lens position with a rotationally asymmetric refractive multifocal intraocular lens. Int J Ophthalmol.

[16] Date RC, Yu F, Miller KM (2013). Confirmation and refinement of the Diehl-Miller nomogram for intraocular lens power calculation after laser in situ keratomileusis. J Cataract Refract Surg.

[17] Feiz V, Mannis MJ, Garcia-Ferrer F, Kandavel G, Darlington JK, Kim E (2001). Intraocular lens power calculation after laser in situ keratomileusis for myopia and hyperopia: a standardized approach. Cornea.

[18] Savini G, Hoffer KJ, Carbonelli M, Barboni P (2010). Intraocular lens power calculation after myopic excimer laser surgery: clinical comparison of published methods. J Cataract Refract Surg.

[19] Einighammer J, Oltrup T, Bende T, Jean B (2009). The Individual Virtual Eye: a Computer Model for Advanced Intraocular Lens Calculation. J Optom.

[20] Haigis W, Goes F. IOL calculation after laser refractive surgery for hyperopia with current measurements. XXVI Congr Eur Soc Cataract Refract Surg ESCRS; Berlin, 2008;2008.

[21] Shammas HJ, Shammas MC, Hill WE (2013). Intraocular lens power calculation in eyes with previous hyperopic laser in situ keratomileusis. J Cataract Refract Surg.

[22] Barrett GD (1993). An improved universal theoretical formula for intraocular lens power prediction. J Cataract Refract Surg.

[23] Savini G, Bedei A, Barboni P, Ducoli P, Hoffer KJ. Intraocular lens power calculation by ray-tracing after myopic excimer laser surgery. Am J Ophthalmol. 2014;157:150–3 e1. 10.1016/j.ajo.2013.08.006 24099275

[24] Wang L, Hill WE, Koch DD (2010). Evaluation of intraocular lens power prediction methods using the American Society of Cataract and Refractive Surgeons Post-Keratorefractive Intraocular Lens Power Calculator. J Cataract Refract Surg.

[25] Francone A, Lemanski N, Charles M, Borboli-Gerogiannis S, Chen S, Robert MC (2019). Retrospective comparative analysis of intraocular lens calculation formulas after hyperopic refractive surgery. PLoS One.

[26] Wu Y, Liu S, Liao R (2017). Prediction accuracy of intraocular lens power calculation methods after laser refractive surgery. BMC Ophthalmol.

[27] Sachdev GS, Sachdev M (2017). Optimizing outcomes with multifocal intraocular lenses. Indian J Ophthalmol.

[28] Savini G, Hoffer KJ (2018). Intraocular lens power calculation in eyes with previous corneal refractive surgery. Eye Vis (Lond).

[29] Ghiasian L, Abolfathzadeh N, Manafi N, Hadavandkhani A (2019). Intraocular lens power calculation in keratoconus; A review of literature. J Curr Ophthalmol.

[30] Savini G, Hoffer KJ, Barboni P (2015). Influence of corneal asphericity on the refractive outcome of intraocular lens implantation in cataract surgery. J Cataract Refract Surg.

[31] Haigis W, Lege B, Miller N, Schneider B (2000). Comparison of immersion ultrasound biometry and partial coherence interferometry for intraocular lens calculation according to Haigis. Graefes Arch Clin Exp Ophthalmol.

[32] Aramberri J (2003). Intraocular lens power calculation after corneal refractive surgery: double-K method. J Cataract Refract Surg.

[33] Hipolito-Fernandes D, Luis ME, Serras-Pereira R, Gil P, Maduro V, Feijao J, et al. Anterior chamber depth, lens thickness and intraocular lens calculation formula accuracy: nine formulas comparison. Br J Ophthalmol. 2020. 10.1136/bjophthalmol-2020-317822 33229347

[34] Pinero DP, Soto-Negro R, Ruiz-Fortes P, Perez-Cambrodi RJ, Fukumitsu H (2019). Interchangeability of corneal curvature and asphericity measurements provided by three different devices. Int J Ophthalmol.

[35] Schiano-Lomoriello D, Bono V, Abicca I, Savini G (2020). Repeatability of anterior segment measurements by optical coherence tomography combined with Placido disk corneal topography in eyes with keratoconus. Sci Rep.

[36] Penna RR, de Sanctis U, Catalano M, Brusasco L, Grignolo FM (2017). Placido disk-based topography versus high-resolution rotating Scheimpflug camera for corneal power measurements in keratoconic and post-LASIK eyes: reliability and agreement. Int J Ophthalmol.

[37] Wang L, Tang M, Huang D, Weikert MP, Koch DD (2015). Comparison of Newer Intraocular Lens Power Calculation Methods for Eyes after Corneal Refractive Surgery. Ophthalmology.

[38] Liu J, Wang L, Chai F, Han Y, Qian S, Koch DD (2019). Comparison of intraocular lens power calculation formulas in Chinese eyes with axial myopia. J Cataract Refract Surg.

